# Genomic Sequence of the WHO International Standard for Hepatitis A Virus RNA

**DOI:** 10.1128/genomeA.00402-18

**Published:** 2018-05-10

**Authors:** Adrian Jenkins, Rehan Minhas, Clare Morris, Neil Berry

**Affiliations:** a Division of Virology, National Institute for Biological Standards and Control, South Mimms, United Kingdom

## Abstract

The World Health Organization (WHO) international standard for hepatitis A virus (HAV) RNA nucleic acid assays was characterized by complete genome sequencing. The entire coding sequence and noncoding regions were assigned HAV genotype IB. This information will aid the design, development, and evaluation of HAV RNA amplification assays.

## GENOME ANNOUNCEMENT

Hepatitis A virus (HAV) is a nonenveloped single-stranded RNA virus with a genome of 7.5 kb and is a member of the Hepatovirus genus within the Picornaviridae family that causes acute liver infection, potentially resulting in HAV disease (1). World Health Organization (WHO) international standards (IS) for HAV RNA represent a means to calibrate secondary viral diagnostic reference materials or can be used directly in the validation of HAV genomic RNA detection assays (2). Elucidation of HAV RNA contamination of plasma pools in the manufacture of blood derivatives is also an essential component of blood safety algorithms to reduce the risk of infection transmission.

The first WHO IS developed for HAV RNA genome amplification techniques (HAV IS 00/562) represents a cornerstone of HAV diagnostic algorithms (2). Replacement preparations, on which the currently used 3rd WHO IS is based, have since been sourced and evaluated, although no genetic composition of any HAV reference preparation has been described. For most purposes, a consensus sequence provides a baseline by which to evaluate assay performance. Hence, we applied Sanger-based sequencing protocols to derive a consensus genome sequence of the 3rd HAV IS that spans all major viral coding regions and noncoding regions, representing ∼98% of the complete HAV genome.

This 3rd HAV IS was prepared from a stock held at the National Institute for Biological Standards and Control (NIBSC) of an HAV-positive plasma donation sourced from the National Plasma Repository, South African National Blood Service, in 2011. To elucidate sequence identity, viral RNA was extracted using total nucleic acid isolation (TNAi) extraction methodologies on an Ampliprep instrument (Roche Ltd.). Rescued RNA was reverse transcribed and directly amplified with the SuperScript III one-step reverse transcription-PCR (RT-PCR) system with Platinum *Taq* HiFi (Invitrogen) using primer pairs described previously (3, 4). In combination, these were as follows: GCCTAGGCTATAGGCTAAAT (HAV-1F) and CGTTCCCAACATCTGTGT (HAV-1R), GTTGTAAATATTAATTCCTGCAGG (HAV-2F) and CAGACAATCCACTTAATGCAT (HAV-2R), CTATGAAGAGATGCTTTGGAT (HAV-3F) and TGTATCTCAATTCCAAATCTTGC (HAV-3R), ATTCATTCTGCTGAYTGGTTG (HAV-4F) and CAACTGGRATAACCTTGATCT (HAV-4R), AGGAAGATTGGAAATCTGATG (HAV-5F) and TTCACTGTTGTAATRCCAACTTG (HAV-5R), CAAGTTGGCATTACAACAGTG (HAV-6F) and GAGCAATTCTATCCATCATTG (HAV-6R), GAAATGGATGCTGGAGTTTTTACT (HAV-7F) and CTGAACARATATCYCTAAGCC (HAV-7R), GTTGAGAGTGATGAATTATGC (HAV-8F) and YTGTCCACTATATCCATCCCA (HAV-8R) and AATGGTGMCAAGATGTGAGCC (HAV-9F) and AACTGCAACCCACTTRTGRTT (HAV-9R), GGGATTATCAGATGATGACAA (HAV-10F) and TACCTCTCCARGCTTGATCAA (HAV-10R), GGACTCCAATGTTAATTTCAG (HAV-11F) and TCCATATTRATTGCATCTATCCC (HAV-11R), GATGAGCCAGATGATTATAAAGA (HAV-12F) and AGAAGGCATTGAMCCACATAC (HAV-12R), GTATGTGGKTCAATGCCTTCT (HAV-13F) and WATTTACTGAAAAGAYAAAATAAACAAAC (HAV-13R), TTCAAGAGGGGTCTCCGG (HAV-1F) and GGAGAGCCCTGGAAGAAAGA (240R), TCACCGCCGTTTGCCTAG (68F) and AGGAATGAGGAAAAACCTAAA (725R), TTATGTGGTGTTTGCCTCTGAG (661F) and AACAAACCATGAGGATAAACT (1249R), ATTTGAGCTAGCGTGCTATGGTACCTGGTGACCA (1180F) and GTTAGAAGGAGAGGTCAATCTG (2115R), GTGAGTACACTGCCATTGG (2050F) and CATTTCCTAGGAGGTGG (3225R), CCAGAGCTCCATTGAACTC (3006F) and AACTGAACAACCAATATCTGC (3908R), CATAATTGGTTTGTTGAGAGTC (3875F) and ATGAATTCAGTCATGTTTTG (4985R), GACTGAATTCATGGAGTTG (4977F) and CATATCAAGATCTAGAAAATCATC (6225R), AACTGTAAATGGAACCCCTAT (5654F) and ATAATTCATCCCACTGTCTATC (6636R), GGGTAAGACTCAGTTAGTTGATG (6225F) and TTGTCCAATCAAATCAAGATTATC (7044R), and TTGTCCAATCAAATCAAGATTATC (6984F) and TTTTTTTTTTTTTTT (15dT).

HAV-specific amplicons were sequenced (ABI PRISM BigDye Terminator kit; Applied Biosystems) using a cycling profile of 25 cycles (96°C for 30 s, 50°C for 15 s, and 60°C for 4 min) on a 3130XL sequencer (ABI Ltd.) in forward and reverse directions and assembled and analyzed using Geneious v7 software. A contiguous sequence was represented by 7,386 nt, with a total GC content of 38.2%. Phylogenetic analyses indicated that the 3rd HAV IS clusters among HAV genotype IB sequences, with no subtype recombination events identified. The complete genome sequence reported here, derived from the 3rd WHO HAV RNA IS, will support genome amplification assay development for HAV RNA detection and quantification in environmental samples and clinical studies.

### Accession number(s).

The genome sequence of the 3rd WHO IS for HAV RNA reported here has been deposited in GenBank under accession number KY003229.
